# Supplementary material for: A cross-reactive human IgA monoclonal antibody blocks SARS-CoV-2 spike-ACE2 interaction
Source: Nat Commun. 2020 Aug 21;11:4198. doi: 10.1038/s41467-020-18058-8 (PMC7442812; doi:10.1038/s41467-020-18058-8)
Supplement: Supplementary file 1 — Supplementary Information [file 41467_2020_18058_MOESM1_ESM.pdf]

Supplemental information

A cross-reactive human IgA monoclonal antibody blocks SARS-CoV-2 Spike-ACE2 interaction

Ejemel et al.

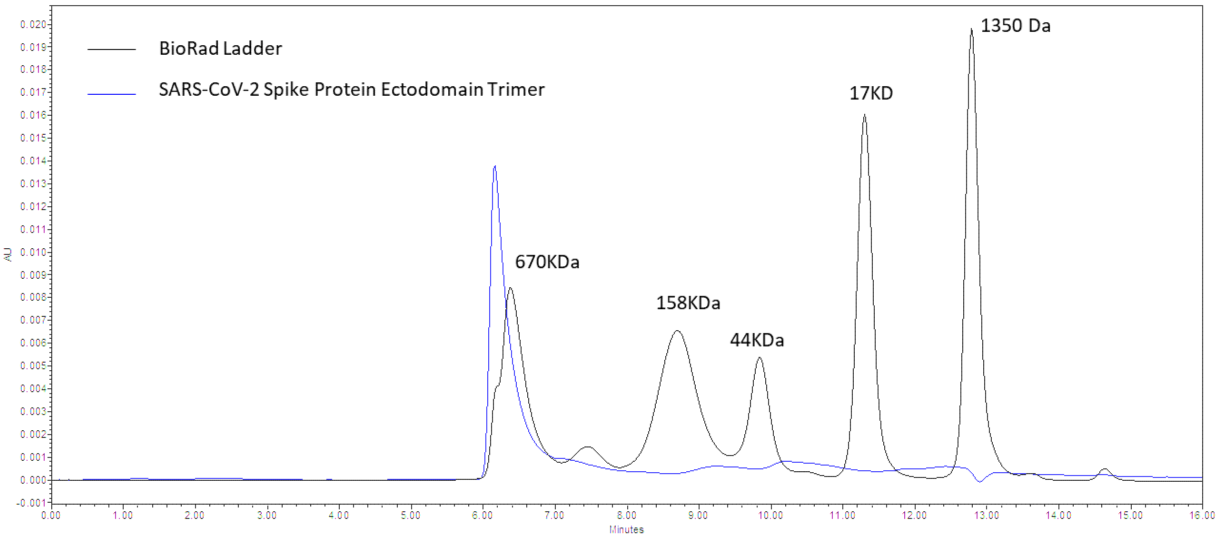

Supplementary Figure 1. Purity of SARS-CoV-2 Ectodomain Trimer by SEC-HPLC.

Spike ectodomain trimer was eluted from SEC-HPLC as a singular peak that aligned with the 670 kDa marker indicating purified intact trimer.

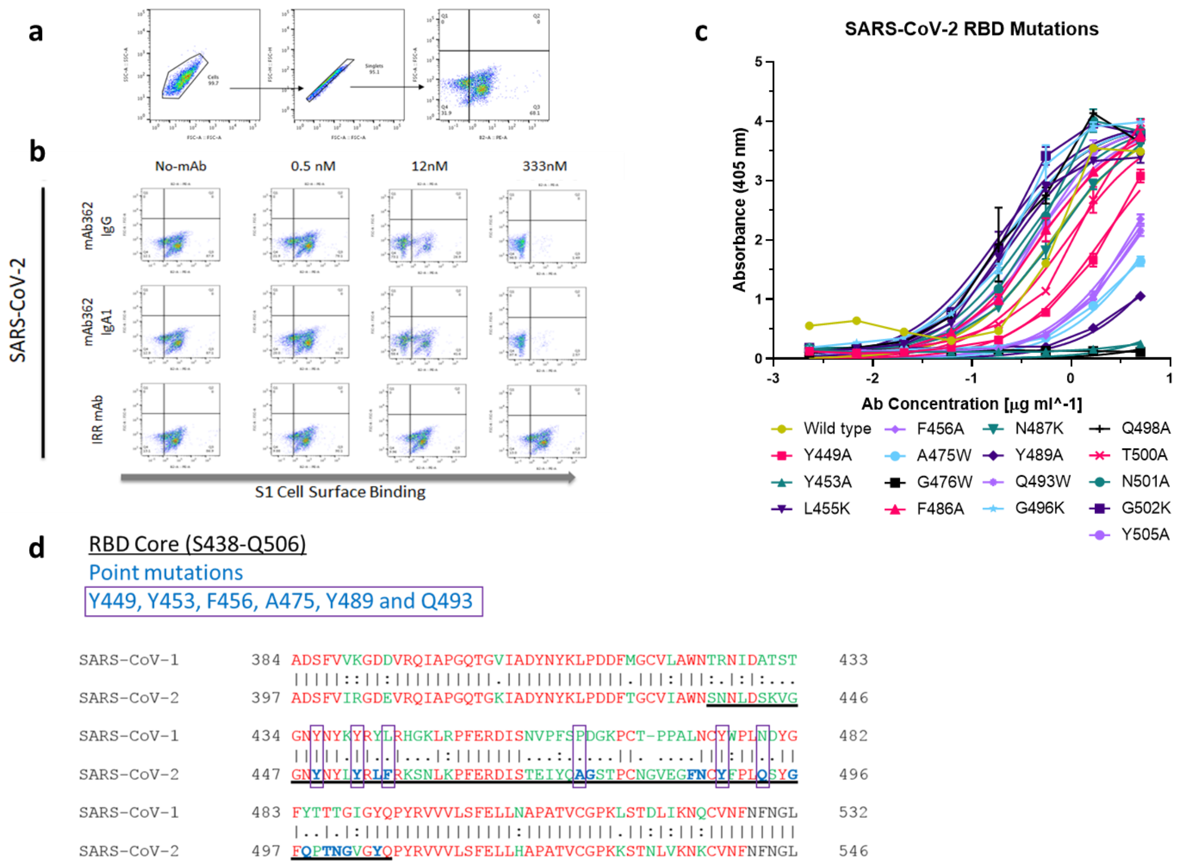

Supplementary Figure 2. MAb362 blocks SARS-CoV and SARS-CoV-2 binding

(a) Flow cytometry gating strategy. Cells were selected in FSC/SSC dot plot, single cells were gated using the FSC-A/FSC-H dot plot. PE+ cells were gated and compared with a control sample with no detectable fluorescence. (b) Flow cytometry of MAb362 IgG and IgA blocking binding of S_1 truncations of the SARS-CoV (S_{1-590}) and SARS-CoV-2 (S_{1-604}) binding to cell surface receptor hACE2 on Vero E6 cells. (c) Binding affinity of wild type (yellow) and fifteen SARS-CoV-2 RBD variants validated critical residues. Data are plotted as the mean ± s.d. from n=3 independent experiments. (d) Sequence alignment show the identified critical residues (Y449, F453, F456, A475, Y489, and Q493) reside within the core domain of the SARS-CoV and SARS-CoV-2 S protein RBD. Source data are provided as a Source Data file.

a

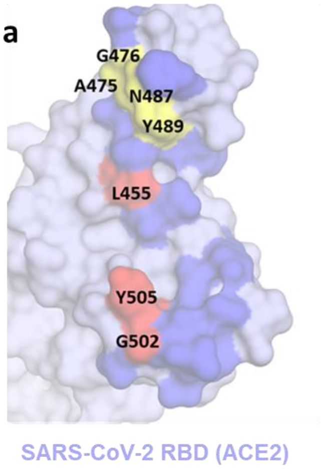

b

	EC$_{50}$ (μg mL^-1)	Fold change to WT
Wild type	2.88	1.00
Y449A	2.95	1.02
Y453A	3.88	1.35
L455K	45.06	15.63
F456A	2.66	0.92
A475W	20.92	7.26
G476W	16.70	5.79
N487K	22.52	7.81
Y489A	25.27	8.77
Q493W	1.19	0.41
G496K	9.66	3.35
Q498A	0.29	0.10
T500A	2.86	0.99
N501A	2.61	0.91
G502K	41.62	14.44
Y505A	33.75	11.71

c

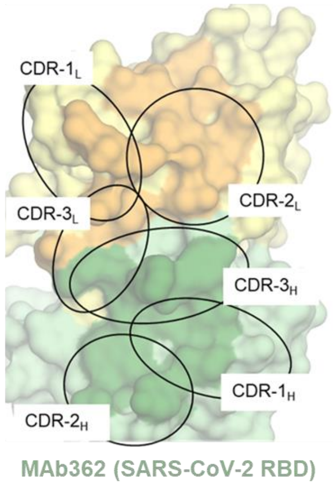

Supplementary Figure 3.

(a) The binding interface on SARS-CoV-2 RBD with hACE2. Key residues identified with effect in mutagenesis are labeled and colored according to influence degree; red represents strongest defects in binding, orange for medium defects and yellow for subtle defects. (b) RBD residues predicted by modeling were expressed as recombinant proteins. ELISAs assay were performed to determine the effect of the mutated residues on RBD binding to hACE2. Key residues (orange) were identified as RBD mutations that reduced EC$_{50}$ values to the wild type RBD (blue). EC$_{50}$ values calculated from n=3 biologically independent experiments. (c) The binding interface on MAb362 with SARS-CoV-2 RBD. The binding interface shown as darker shade is defined as having vdW contacts greater than -0.5 kcal mol^-1. Residues from all CDR's from both heavy and light chains pack against the SARS-CoV-2 RBD are labeled by circles.

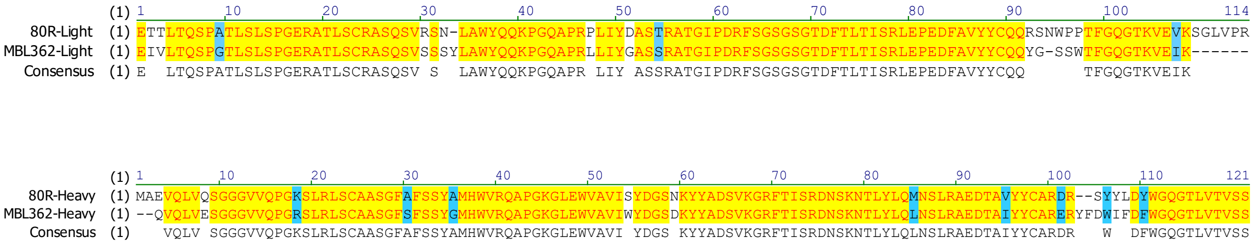

Supplementary Figure 4. Alignment of MAb362 heavy and light chain with 80R.

Amino acid sequence alignment of the variable regions of MAb362 and 80R heavy and light chains.

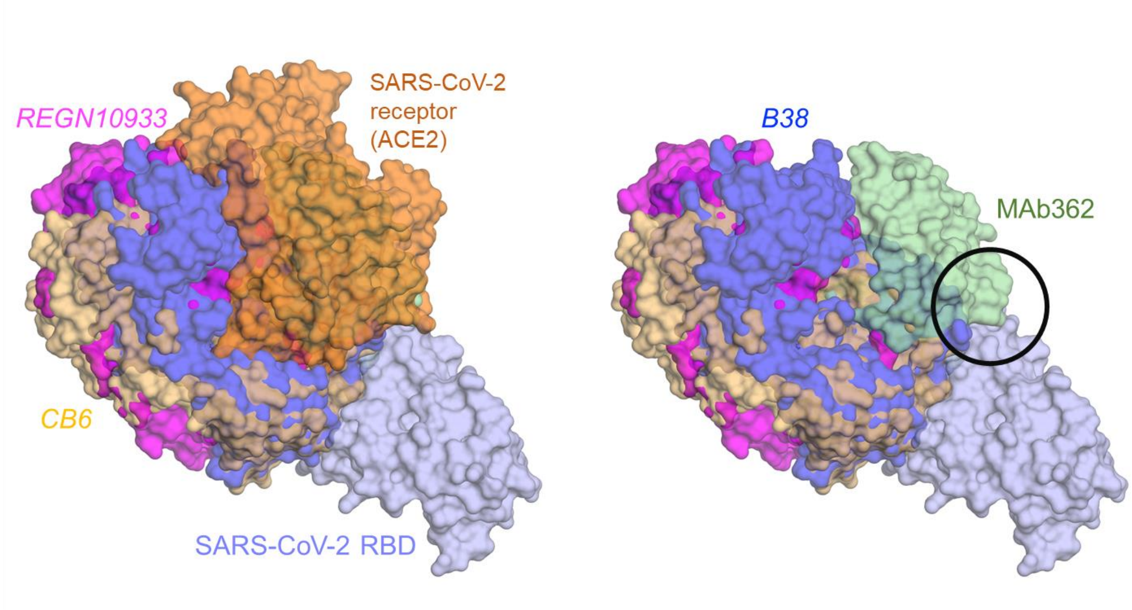

Supplementary Figure 5. Superposition of published SARS-CoV-2 RBD neutralizing antibodies that bind to hACE2 interface.

Supposition of MAb362 (green) compared to other published SARS-CoV-2 RBD (violet) neutralizing antibodies that bind to hACE2 (orange) interface, REGN10933 (magenta), CB6 (wheat) and B38 (blue). MAb362 blocks hACE2 binding interface in a conserved region different from other antibodies identified to date.

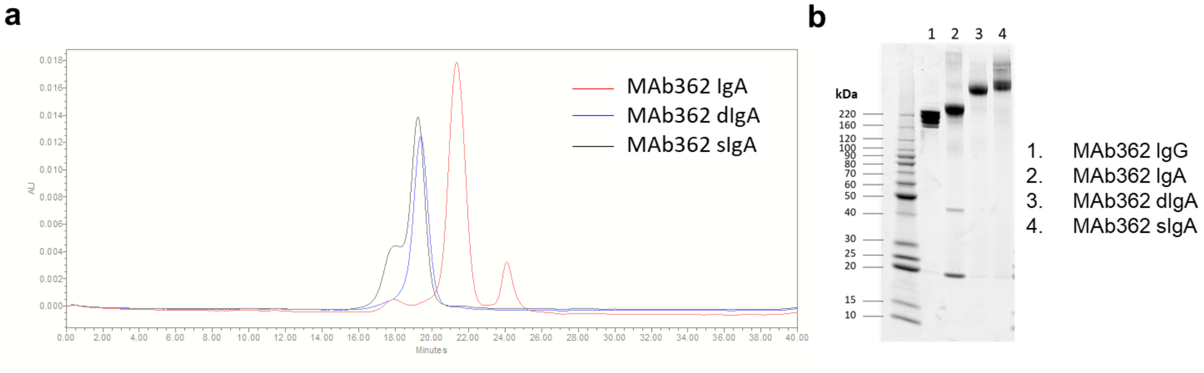

Supplementary Figure 6. Purified MAb362 isotypes.

(a) MAb362 IgA (red), dIgA (blue), and sIgA (black) were purified by size exclusion chromatography by FPLC and analyzed in HPLC as described in the method. The HPLC profile is shown as isotypes detected at different time points according to their molecular sizes. (b) Purified MAbs were then run on SDS-PAGE followed by Ruby staining to confirm the expected molecular weights for IgG, IgA, dIgA, and sIgA. This gel is representative of n>3 independent experiments. Source data is in Source Data File.

Clone	Epitope (A.A.)	SARS-CoV S1 S_{1-509}	SARS-CoV RBD $S_{270-510}$	SARS-CoV-2 S1 S_{1-604}	SARS-CoV-2 RBD $S_{319-541}$
7-508-395	90-190	>0.5	-	-	-
7-508-16	130-150	>0.5	-	-	-
7-508-39	130-150	>0.5	-	-	-
7-508-68	130-150	>0.5	-	-	-
7-508-104	130-150	>0.5	-	-	-
7-508-415	130-150	>0.5	-	-	-
7-508-466	370-510	>0.5	>0.5	-	-
12-28-1	490-510	>0.5	>0.5	-	-
7-73-121	490-510	>0.5	>0.5	-	-
7-508-201	490-510	>0.5	>0.5	-	-
7-508-669	490-510	>0.5	>0.5	-	-
7-508-362	N/A	>0.5	>0.5	>0.5	>0.5
7-508-94	N/A	>0.5	>0.5	-	-
7-508-411	N/A	>0.5	>0.5	-	-
7-508-478	N/A	>0.5	>0.5	-	-
7-508-568	N/A	>0.5	>0.5	-	-
7-509-2	N/A	>0.5	>0.5	-	-
7-508-73	N/A	>0.5	-	-	-
7-508-19	N/A	>0.5	-	-	-
7-508-165	N/A	>0.5	-	-	-
7-508-254	N/A	>0.5	-	-	-
7-508-223	N/A	>0.5	-	-	-
7-508-315	N/A	>0.5	-	-	-
7-508-528	N/A	>0.5	-	-	-
7-508-592	N/A	>0.5	-	-	-
7-512-9	N/A	>0.5	-	-	-
7-508-143	N/A	-	0.1 - 0.5	-	-
7-508-198	N/A	0.1 - 0.5	0.1 - 0.5	-	-
7-508-380	N/A	0.1 - 0.5	>0.5	-	-
12-28-4	N/A	-	-	-	-
7-73-57	N/A	-	-	-	-
7-508-448	N/A	-	-	-	-
7-508-573	N/A	-	-	-	-
7-508-646	N/A	-	-	-	-
7-508-680	N/A	-	-	-	-
7-508-694	N/A	-	-	-	-

Supplementary Table 1. Screening of a Panel of SARS-CoV MAbs for Cross-Binding Activity. A panel of 36 previously generated frozen hybridomas of anti-SARS-CoV MAbs were recovered and scaled up. Hybridoma supernatants were screened for reactivity to the SARS-CoV-2 S protein. Binding for each truncation is reported as absorbance at 450 nm for hybridoma supernatant. Positive cell clones were selected for antibody sequencing.
