# Supplementary material for: A cross-reactive human IgA monoclonal antibody blocks SARS-CoV-2 spike-ACE2 interaction
Source: Nat Commun. 2020 Aug 21;11:4198. doi: 10.1038/s41467-020-18058-8 (PMC7442812; doi:10.1038/s41467-020-18058-8)
Supplement: Supplementary file 3 — Reporting Summary [file 41467_2020_18058_MOESM3_ESM.pdf]

nature research

Corresponding author(s): Yang Wang, Celia Schiffer

Last updated by author(s): July 17, 2020

Reporting Summary

Nature Research wishes to improve the reproducibility of the work that we publish. This form provides structure for consistency and transparency in reporting. For further information on Nature Research policies, see our Editorial Policies and the Editorial Policy Checklist.

Statistics

For all statistical analyses, confirm that the following items are present in the figure legend, table legend, main text, or Methods section.

n/a	Confirmed	
☐	☒	The exact sample size (*n*) for each experimental group/condition, given as a discrete number and unit of measurement
☐	☒	A statement on whether measurements were taken from distinct samples or whether the same sample was measured repeatedly
☐	☒	The statistical test(s) used AND whether they are one- or two-sided<br>*Only common tests should be described solely by name; describe more complex techniques in the Methods section.*
☒	☐	A description of all covariates tested
☒	☐	A description of any assumptions or corrections, such as tests of normality and adjustment for multiple comparisons
☐	☒	A full description of the statistical parameters including central tendency (e.g. means) or other basic estimates (e.g. regression coefficient) AND variation (e.g. standard deviation) or associated estimates of uncertainty (e.g. confidence intervals)
☒	☐	For null hypothesis testing, the test statistic (e.g. *F*, *t*, *r*) with confidence intervals, effect sizes, degrees of freedom and *P* value noted<br>*Give P values as exact values whenever suitable.*
☒	☐	For Bayesian analysis, information on the choice of priors and Markov chain Monte Carlo settings
☒	☐	For hierarchical and complex designs, identification of the appropriate level for tests and full reporting of outcomes
☒	☐	Estimates of effect sizes (e.g. Cohen's *d*, Pearson's *r*), indicating how they were calculated

Our web collection on statistics for biologists contains articles on many of the points above.

Software and code

Policy information about availability of computer code

Data collection	MACSQuantify Software v2.11<br>Modeller (https://salilab.org/modeller/)<br>Schrödinger (https://www.schrodinger.com/).
Data analysis	GraphPad Prism v8.1.1<br>Fortebio Data Analysis v8.1<br>FlowJo v10<br>Softmax Pro v4.3.1 LS<br>PyMOL Molecular Graphics System v2.3.4<br>Glide, Bioluminate, and Desmond are applications in Schrödinger suite v19-4<br>Modeller, v9.23<br>Microsoft Excel v2016<br>R version v3.5.3 (2019-03-11)<br>Library ggplot2 v3.3.0 (for graph plotting)<br>The script used to calculate the mid-point probit is from this reference:<br>R. M. Johnson, L. Dahlgren, B. D. Siegfried, M. D. Ellis, Acaricide, fungicide and drug interactions in honey bees (Apis mellifera). PLoS One 8, e54092 (2013).

For manuscripts utilizing custom algorithms or software that are central to the research but not yet described in published literature, software must be made available to editors and reviewers. We strongly encourage code deposition in a community repository (e.g. GitHub). See the Nature Research guidelines for submitting code & software for further information.

Data

Policy information about availability of data

All manuscripts must include a data availability statement. This statement should provide the following information, where applicable:

- Accession codes, unique identifiers, or web links for publicly available datasets
- A list of figures that have associated raw data
- A description of any restrictions on data availability

Antibody sequences are available at GenBank (accession number MT789771 and MT789772). PDB files used in the study include: PDB 2GHW, the complex of 80R:SARS-CoV-RBD24; PDB 2AJF, the complex of ACE2:SARS-CoV-RBD43 and PDB 6VW1, the complex of ACE2:SARS-CoV-2-RBD. All other data generated are included in figures and tables in this published article.

Field-specific reporting

Please select the one below that is the best fit for your research. If you are not sure, read the appropriate sections before making your selection.

☒ Life sciences ☐ Behavioural & social sciences ☐ Ecological, evolutionary & environmental sciences

For a reference copy of the document with all sections, see nature.com/documents/nr-reporting-summary-flat.pdf

Life sciences study design

All studies must disclose on these points even when the disclosure is negative.

Sample size	No sample size calculation was performed to design the study because one antibody from one hybridoma is studied.
Data exclusions	Data were not excluded from analysis
Replication	All assays were conducted with at least n=2 independent runs. Results between multiple independent runs were consistent and successfully replicated.
Randomization	Randomization is not applicable in this study because one antibody from one hybridoma is studied.
Blinding	Blinding is not relevant for this study because it is not a case control study.

Reporting for specific materials, systems and methods

We require information from authors about some types of materials, experimental systems and methods used in many studies. Here, indicate whether each material, system or method listed is relevant to your study. If you are not sure if a list item applies to your research, read the appropriate section before selecting a response.

Materials & experimental systems

n/a	Involved in the study	
☐	☒	Antibodies
☐	☒	Eukaryotic cell lines
☒	☐	Palaeontology and archaeology
☒	☐	Animals and other organisms
☒	☐	Human research participants
☒	☐	Clinical data
☒	☐	Dual use research of concern

Methods

n/a	Involved in the study	
☒	☐	ChIP-seq
☐	☒	Flow cytometry
☒	☐	MRI-based neuroimaging

Antibodies

Antibodies used	1. Alkaline phosphatase affiniPure goat anti-Human IgG, Jackson ImmunoResearch (109-055-098) 1:1000 for ELISA.<br>2. Alkaline phosphatase affiniPure goat anti-Mouse IgG, Jackson ImmunoResearch (115-055-003) 1:1000 for ELISA.<br>3. Horseradish peroxidase-conjugated anti-kappa, Cat 2060-05, Company Southern biotech, 1:2,000 (For ELISA) 1:20000 for ELISA<br>4. Phycoerythrin-conjugated anti-mouse IgG, Jackson ImmunoResearch (115-116-071) 1:20 for the flow cytometry.<br>5. Mouse-anti-Myc antibody, BD Pharmingen (551101), 1:100 for flow cytometry<br>6. Horseradsih peroxidase-conjugated goat anti-mouse, Jackson ImmnuoResearch (115-035-062), 1:2000 for ELISA.
Validation	Primary antibodies have been validated by different assays including ELISA and SDS-PAGE. Commercial secondary antibodies were purchased from vendors and were validated by the manufacturers.

Eukaryotic cell lines

Policy information about cell lines

Cell line source(s)	1. African green monkey origin, Vero-E6, from ATCC (ATCC CRL-1586)<br>2. Human embryonic kidney cell, HEK293T, from ATCC (ATCC CRL-3216)
Authentication	Cell lines have been authenticated by ATCC using STR profiling.
Mycoplasma contamination	Cells were tested as mycoplasma negative.
Commonly misidentified lines<br>(See ICLAC register)	None were used.

Flow Cytometry

Plots

Confirm that:

- [x] The axis labels state the marker and fluorochrome used (e.g. CD4-FITC).
- [x] The axis scales are clearly visible. Include numbers along axes only for bottom left plot of group (a 'group' is an analysis of identical markers).
- [x] All plots are contour plots with outliers or pseudocolor plots.
- [x] A numerical value for number of cells or percentage (with statistics) is provided.

Methodology

Sample preparation	Vero E6 cell was obtained from ATCC. In brief, cells were incubated with SARS-CoV and SARS-CoV-2 S1 protein and followed by mouse anti-Myc primary antibody and goat anti-mouse IgG-PE secondary antibody as stated in the method part.
Instrument	Flow cytometry was performed using MACSQuant Analyzer 10 Flow Cytometer (Miltenyi Biotec)
Software	Flow cytometry data was collected with MACSQuantify Software v2.11 and analyzed with FlowJo V10
Cell population abundance	No sorting was performed with the flow cytometer
Gating strategy	FSC/SSC gates were used to select mononuclear cells. Control antibody staining was used to define positive/negative cell populations.

- [x] Tick this box to confirm that a figure exemplifying the gating strategy is provided in the Supplementary Information.
